# Supplementary material for: Attentional efficiency does not explain the mental state × domain effect
Source: PLoS One. 2020 Jun 15;15(6):e0234500. doi: 10.1371/journal.pone.0234500 (PMC7295218; doi:10.1371/journal.pone.0234500)
Supplement: Attachment — Submitted filename: PLoS ONE Response to reviewers.docx [file pone.0234500.s001.docx]

**Response to reviewers (PONE-D-20-11124)**

**Editor comments**

*Moreover, while reading, I had a thought which I am not sure how relevant is (but it should be). In a paper with Jim Everett and Brian Earp, we also found some evidence of "moral pluralism" using something similar (I think) to a mental state x moral domain effect, although in a different context. As scale we had the Oxford Utilitarianism Scale (Kahane et al., Psych Rev, 2018), which measures how utilitarian a subject is along two dimensions, instrumental harm and impartial beneficence; half of the participants took this scale when primed to follow their emotion and the other half when primed to follow their reason. We found that priming emotion decreases instrumental harm, while leaving impartial beneficence unaffected (Capraro, Everett, & Earp, J Exp Soc Psych, 2019). It seems related to your point (mental state seen as system 1 vs system 2 and moral domain seen as impartial beneficence vs instrumental harm), but I am not super sure. So, feel free to ignore this comment if you feel it is unrelated.*

Response: Thanks for letting us know about this interesting work. There are some peripheral similarities (different effects across responses to different types of moral stimuli) with our work. However, it is unclear to me just where “impartial beneficence” fits into popular accounts of moral pluralism (e.g., moral foundations theory). Having looked at your paper it seems that it is a mix of justice and harm concerns. Looking at responses to stimuli that represent multiple moral domains is an interesting general avenue for future work, as is examining how utilitarianism and dual process accounts map on to moral pluralism, but we feel this is beyond the scope of the present paper. Here we have a specific focus on explanations of the classic mental state x domain effect.

**Reviewer 1**

This reviewer raised four issues:

Issue 1: *On page 4, the authors write that “convergent evidence has been found both in fMRI and cross-cultural work”. I recommend they describe the convergent evidence from these studies in some detail.*

Response: We have now included additional details of the converging evidence for the mental state × domain interaction (p.4).

Issue 2: *The authors write that “the weirdness of purity (vs. harm) scenarios may compete with standard moral cognition for attentional resources” (p5). Has this general effect of weirdness on attentional resources ever been tested directly in prior studies? If so, the authors should cite studies that provide evidence of this relationship.*

Response: To our knowledge, this has not been directly tested in terms of “weirdness” but a fair amount of work has shown that surprising and unexpected stimuli, which we argue purity violations like incest and drinking urine are clear cases of, compete for attentional resources and can lead to “surprise-induced blindness” (analogous to attentional blink) in attentional processes. We have now added details of this (p5).

Issue 3: *The authors speculate on p15 that “if the mental state × domain effect is driven by the weirdness of the purity vignettes making mental state information less salient, then making mental state information highly salient should “knock out” the mental state × domain effect.” This hypothesis was recently tested in an fMRI study: Dungan & Young (2019), SCAN. The authors found that prompting participants to focus on intent information did not knock out the mental state x domain effect.*

Response: We have now added discussion of this study (p15). Specifically, we suggest that more “bottom-up” (e.g., directly manipulating stimulus salience) approaches could prove more effective than “top-down” manipulation of task goals for manipulating the salience of mental state information.

Issue 4: *The section on page 15, beginning with “Indeed, such methods…” and ending with “measure of attentional processes like the ANTs”, is a bit unclear. It would help if the authors could explain what it would mean to make decision prompts contingent on eye movements in the context of a moral judgment task.*

Response: We have briefly sketched out how one might do this (p16-17).

This reviewer also was kind enough to point out some typos/grammatical errors:

*Typos:*

*1) Cue is mis-spelled que in several places.*

*2) There are a few grammatical errors in this sentence:*

*Another added benefit of such methods is that they may also afford testing of manipulation-of-mediator and measurement-of-mediation designs [23], something that makes little theoretically sense for individual difference measure of attentional processes like the ANT [24].*

Response: We have corrected these typos and grammatical errors and are grateful to the reviewer for spotting them.

**Reviewers 2a and 2b**

These reviewers raised over a dozen issues. We have gone through these with careful thought and acted on them where we believed that it would make for a better manuscript:

Issue 1: *In the early parts of the introduction I would have appreciated seeing a more thorough definition of what is meant by ‘harm’ versus ‘purity’ violations. Examples are provided, but not explanations of the particular characteristics of each domain. As a result, readers who are not familiar with the moral foundations might see the narrow examples provided in the study as fully representative of the relevant domains. As your paper has one study and is thus not too long, I would like to see a more comprehensive introduction including the specific points in the following comments. PLOS readers tend to be multi-disciplinary and will need more fleshed out introductions to these areas of research.*

Issue 2: *On page 3, the examples provided for each “domain of action” are all violations – not all moral actions are ones that violate, so I would suggest introducing your examples as violations or providing examples that represent virtue and vice.*

Response: We have added more details on this (p3), but we have balanced this with keeping our paper short and focused on testing attentional explanations of the mental state x domain effect. There are ample citations to moral foundations theory in the paper, including lengthy review articles which interested readers can consult if they are interested in the considerable scope of moral foundations theory.

Issue 3: *The introduction should also include a brief section on discrete moral emotions, an area that has contributed to the moral pluralism debate, see Cameron, Lindquist, & Gray (2015) in PSPR and the studies they review in their paper.*

Issue 4: *When introducing moral pluralism, include a brief discussion of the critiques – mainly Kurt Gray and colleagues’ work that everything essentially boils down to harm – even purity violations.*

Response: Examining the role of emotion and the degree to which harm-based “moral templates” can explain evidence for moral pluralism is an interesting topic but given that emotion and Gray’s “harm pluralism” played little role in our theorising or our empirical work we think that reviewing this work will only detract from reporting a theoretically focused and carefully conducted study. To be clear, we tested whether attentional failure could account for the mental state x domain effect. If we had (counterfactually) found that it did, this would not support the idea that our moral cognition for purity violations is based on a harm-based moral template. It would only suggest that key evidence for moral pluralism (i.e., separate, domain-specific moral systems) is better explained by an attentional account. Again, introducing emotion and Gray’s harm pluralism into the introduction of a paper not testing emotion or Gray’s harm-based account, or suggesting any future directions or implications for these approaches seems to take us beyond the scope of our paper and only detracts from the key theoretical contribution of the paper: attentional failure does not explain the mental state x domain effect.

Issue 5: *On page 4 under the ‘Moral systems or attentional failures’ heading the authors introduce the central argument for an alternative explanation to domain-specific moral systems, namely, that purity scenarios depict violations that are “weirder” and less severe than cases of harm. Again, I would have preferred to see more clarifying detail on what is meant by ‘weirdness’ and ‘severity’ and further explanation of how cited authors – and the authors of the manuscript – claim that this influences intention effects.*

Response: We’ve added extra detail on weirdness and severity (p4). In addition, we have added extra details of how weird or unexpected stimuli (like purity violations) may compete for attentional resources, thus making mental state information less salient (see response to Reviewer 1, issues 2; p5).

Issue 6: *On page 4, change vs, to v. at bottom of page*

Response: We’ve corrected this typo and thank the reviewers for spotting it.

Issues 7: *p. 6. The relationship between attentional control and integration of mental state information is presented as a ‘given’, however I would have like this to be explained or justified further. What reasons are there to think that integration of mental state information is affected by attention control in ways that other aspects of moral cognition are not?*

Response: We provide additional citations (p. 7) for work in mental state reasoning/theory of mind (ToM) that demonstrates that attentional control is employed in theory of mind tasks that involve integrating mental state information with other types of information. This is widely excepted in the ToM literature, forming a large part of discussions as to which tasks use “purer” measures of ToM. We do not provide reasons for thinking attentional control plays no role in other aspects of moral cognition because we do not think that is likely to be the case. The only reason we focus on the possible role of attentional control in mental state information is because we are trying to test possible attentional explanations for an effect that is based on differential use of mental state information across moral domains. We think this is now clearer with this and other changes that we have made.

Issue 8: *In the ANT section of Materials and measures, the authors have used two different spellings of ‘cue’.*

Response: We’ve corrected this typo and thank the reviewers for spotting it (see Reviewer 1 Typos).

Issue 9: *I wonder whether the selection of the incest scenario for the purity violation may have confounded the intention-accident distinction. People feel so strongly disgusted by incest, and this scenario has probably been labelled as one of the weirdest in the purity set – perhaps less so than the ones about eating your dead dog. Can you include something to justify how the incest and poisoning vignettes are matched.*

Response: The original Young & Saxe (2011) paper that employed this pair of scenarios empirically examined (Experiment 2) whether the difference in moral judgments of harm versus purity violations was due to differences in the emotional salience of harm vs. purity (i.e., incest) scenarios or the agent’s mental state (intentional vs. accidental). They found no support for these potential confounds. It’s beyond the scope of our paper to address confounds that have already been addressed some 10 years ago. We’d suggest publishing the review history for those that might be thinking the same as the reviewers. This is a good way of allowing a paper to remain concise and focused, without having to address thoughts that have been previously addressed in earlier work.

Issue 10: *Regarding the coding error and the failure to record participant gender and age (need a comma after coding error in the footnote), can you include a note to dismiss any concerns – assuming that you did not expect to find any individual differences based on gender or age.*

Response: We have added to the footnote to address this.

Issue 11: *I could not find where the coding for mental state or domain was recorded, making it difficult to interpret the direction of effects in the models. Similarly, descriptive statistics do not seem to have been reported.*

Response: We are grateful to the reviewers for spotting this and have added full details of the coding to the reformatted regression tables (p 11, 14). We have also added details of the descriptives in a “Supplementary Descriptives” file on the osf page and have directed readers to this (p11). Of course all the data is available of the osf page so anybody can examine, explore, and test any ideas they may have when reading the paper – on of the advantages of open science!

Issue 12: *Given that the central premise of the paper is that the ‘weirdness’ of purity violations interferes with attention that may otherwise be paid to intentionality I would have liked this ‘weirdness’ to be investigated as a variable independent of domain. As Gray and Keeney (2015) have shown, it is possible to conceive of purity violations that are not ‘weird’ and harm violations that are. Without such investigation I feel it is difficult to generalise the intentionality effect equally to all purity violations. This should be mentioned in your limitations and areas for future research.*

Response: We have addressed this in our discussion of limitations and future directions (p17).

Issue 13: *The authors base some of their theorising on Huebner, Dwyer, & Hauser’s (2009) finding that emotion may impact moral judgments through its impact on attention processes. As an alternate explanation for their results, have the authors considered the possibility that both the heightened emotional power of the harm vignettes and the weirdness of the purity vignettes similarly co-opt cognitive processing?*

Response: No, we have not considered this and are not exactly sure what it would mean. If we understand the reviewers correctly, then it would seem to suggest that the emotional saliency of harmful acts and the weirdness of purity acts should both lead to attentional failures. We have empirical reasons to doubt the former as we detailed in our response to issue 9: “The original Young & Saxe (2011) paper that employed this pair of scenarios empirically examined (Experiment 2) whether the difference in moral judgments of harm versus purity violations was due to differences in the emotional salience of harm vs. purity (i.e., incest) scenarios or the agent’s mental state (intentional vs. accidental).” Indeed, weirdness is the only potential confound that we have empirical reasons to think just might drive any attentional effect, which is why we chose to focus on it and its potential impact on attentional processing.
